# Validation of a diagnostic probability function for estimating probabilities of acute coronary syndrome

**DOI:** 10.1186/1471-227X-14-23

**Published:** 2014-11-18

**Authors:** Lukas Zimmerli, Johann Steurer, Reto Kofmehl, Maria M Wertli, Ulrike Held

**Affiliations:** Division of Internal Medicine, University Hospital Zurich, Zurich, Switzerland; Horten Centre for Patient Oriented-Research and Knowledge Transfer, University of Zurich, Pestalozzistrasse 24, Zurich, 8091 Switzerland

**Keywords:** Acute coronary syndrome, Validation study, Risk prediction, Emergency room

## Abstract

**Background:**

We recently reported about the derivation of a diagnostic probability function for acute coronary syndrome (ACS). The present study aims to validate the probability function as a rule-out criterion in a new sample of patients.

**Methods:**

186 patients presenting with chest pain and/or dyspnea at one of the three participating hospitals’ emergency rooms in Switzerland were included in the study. In these patients, information on a set of pre-specified variables was collected and a predicted probability of ACS was calculated for each patient. Approximately two weeks after the initial visit in the emergency room, patients were contacted by phone to assess whether a diagnosis of ACS was established.

**Results:**

Of the 186 patients included in the study, 31 (17%) had an acute coronary syndrome. A risk probability for ACS below 2% was considered a rule-out criterion for ACS, leading to a sensitivity of 87% and a specificity of 17% of the rule. The characteristics of the study patients were compared to the cases from which the probability function was derived, and considerable deviations were found in some of the variables.

**Conclusions:**

The proposed probability function, with a 2% cut-off for ruling out ACS works quite well if the patient data lie within the ranges of values of the original vignettes. If the observations deviate too much from these ranges, the predicted probabilities for ACS should be seen with caution.

**Electronic supplementary material:**

The online version of this article (doi:10.1186/1471-227X-14-23) contains supplementary material, which is available to authorized users.

## Background

The swift and correct diagnosis about the presence/absence of acute coronary syndrome (ACS) in patients with chest pain and/or dyspnea is a major diagnostic challenge. Chest pain accounts for 2-5% of all admissions to the emergency room [1]. ACS is present in about 15-30% of the admitted patients and is the major concern for both doctors and patients [1]. Recently we reported on the derivation of a diagnostic probability function to calculate the probability of an ACS in patients with acute onset of chest pain and/or dyspnea after a first examination, including electrocardiography (ECG) and measurement of troponin [2].

The diagnostic probability function has been derived with an innovative method by garnering tacit experts’ knowledge. To this end 80 hypothetical cases, most of them with a presumably low probability of ACS, were specified and sent to 32 experts. All hypothetical cases described patients with acute onset of chest pain and/or dyspnea, with regard to other relevant diagnostic indicators (e.g. duration of symptoms, quality of chest pain, risk factors) and each case was different. The experts were requested to estimate for each case the probability for the presence of ACS. The medians of the experts’ probabilities were translated into a diagnostic probability function, details of the derivation process can be found elsewhere [2]. The final prediction model is based on shrinked regression coefficients in order to reduce the predictor variables’ weights.

Before applying such a probability function in daily practice its discriminative ability and calibration should be evaluated in a set of true patients with acute onset of chest pain and/or dyspnea. The aim of this study was to assess the diagnostic performance of the risk prediction rule applied to patients in an emergency room setting.

## Methods

The ethics committees of Zurich and St. Gallen approved the study protocol, and we obtained informed consent from all participants.

### Recruitment of patients

Senior physicians of the emergency rooms from the University Hospital Zurich, Waid-City-Hospital Zurich and the Cantonal Hospital in St. Gallen were asked to recruit patients and they declared their willingness to enroll patients in this validation study.

Eligible were patients older than 18 years with the chief complaint of acute onset of chest pain and/or dyspnea attending the emergency room and in which an ECG was recorded and blood drawn for measurement of troponin levels. Only patients were included proclaiming their willingness to answer questions about their health status two weeks after the visit in the emergency room by telephone interview. Patients in need for urgent procedures because of hemodynamic instability were not included.

### Data collection

All patients admitted to the emergency room that were potentially eligible for the study were asked to participate. Study-participation did not delay urgent diagnostic testing or treatment and had no influence on diagnostic and/or treatment decisions. After obtaining informed consent the emergency physician collected information from medical history, performed a physical exam, recorded an ECG and ordered blood tests. All information was recorded about the particulars of the episode (e.g., time since onset of symptoms, duration of symptoms, type of symptoms), associated symptoms during or in days prior to the episode (e.g. nausea, dizziness, fever), possible prompters of the episode (e.g., physical exertion, unusual emotional stress, cocaine use), risk for ACS (e.g., age, smoking, history of hypertension), physical examination (e.g., heart rate, blood pressure, chest pain aggravation by pressure on chest), findings in ECG (e.g., Q-waves, maximum or ST-elevation/depression, hyperacute T-wave) and enzyme levels (troponin). The data were entered in a prepared paper-form questionnaire (shown in Additional file 1) and then transferred into a FileMaker Database (FileMaker Inc., 5201 Patrick Henry Drive, Santa Clara, CA 95054).

Other diagnostic procedures and the decision to initiate treatment or referral of the patient to the intensive care unit or catheter lab were left to the discretion of the treating physician. During the consultation in the emergency room the probabilities of an acute coronary syndrome were not computed to avoid any influence of a probability estimate on the further management of the patient. From all patients hospitalized in the hospital of attendance or referred to another hospital information about the course of the illness was collected, in particular, whether ACS was diagnosed by the treating physician or not. Patients who were sent home after ruling out ACS in the emergency room were contacted again between 10 and 14 days after the consultation in the emergency room by a research assistant by phone. The patients were asked whether they had visited a physician or an emergency room or had been hospitalized within the last two weeks with further episodes of chest pain and/or dyspnea since discharge from the emergency room. In case of uncertainty about the presence of an ACS the family physician was contacted. In patients without ACS no further information about the underlying illness causing chest pain or dyspnea were collected.

### Reference standard

Myocardial infarction and acute coronary syndrome was defined according to the recommendations from the European Society for Cardiology and the American Heart Association [3, 4].The definitions for an acute myocardial syndrome included the detection of rise and/or fall of cardiac biomarkers with at least one value above the 99^th^ percentile of the upper reference limit together with evidence of myocardial ischemia with at least one of the following: symptoms of ischemia, ECG changes indicative of new ischemia (new ST-T changes or left bundle branch block), development of pathological Q waves in the ECG, imaging evidence of new loss of viable myocardium or new abnormality. Acute myocardial ischemia was defined as a new ST elevation at the J-point into contiguous leads with the cut-off point ≥0.2 mV in men or ≥0.15 mV in women in leads V2-3 and/or ≥0.1 mV in other leads. Unstable angina/non-ST-elevated myocardial infarciation (NSTEMI) was defined as constitutes a clinical syndrome with electrocardiogram (ECG) ST-segment depression or prominent T-wave inversion and/or positive biomarkers of necrosis (e.g., troponin) in the absence of ST-segment elevation and in an appropriate clinical setting (chest discomfort or angina equivalent).

The probabilities of ACS were calculated following the published prediction rule, only after all patient data, including information on presence or absence of ACS had been collected.

### Analysis of data

The characteristics of the participating patients are summarized with medians and inter-quartile ranges for the continuous variables, and with percentages for the binary variables. The published risk prediction model is based on 43 variables and some of the original variables measured in the patients needed a transformation according to the procedure described in the derivation study [2]. The variables time since onset of symptoms (hours) and duration of symptoms (minutes) were log-transformed and squared. The number of cigarettes smoked per day in the days prior to the episode, the number of pack years reduced by 10, the age, the heart rate and the body mass index were squared. The mean blood pressure was calculated as arithmetic mean between systolic and diastolic blood pressure. Two product terms needed to be calculated, these were the log-transformed time since onset of symptoms x ST elevation and log-transformed time since onset of symptoms squared x ST elevation. The percentage of missing data was assessed and missing observations were multiply imputed (m = 5) where necessary. We used partial mean matching for the continuous variables, and logistic regression for the binary variables for the imputation, based on all variables in the dataset. When all variables were in the correct form, the shrinked regression coefficients of the prediction model were applied to the data of all patients in the study, resulting in an estimated probability of ACS for each individual. The final predicted probabilities were calculated as arithmetic mean of the five imputations. These probabilities are summarized with median and range. We dichotomized the predicted probabilities with a cut-off at <2% as a rule-out criterion for ACS according to MacGougan et al. [5]. We present the results of the binary diagnostic rule for ACS with respect to its sensitivity and specificity, positive and negative predictive values, and positive and negative likelihood ratios. All analyses were performed with R statistical software for windows [6].

## Results

**Figure 1:**
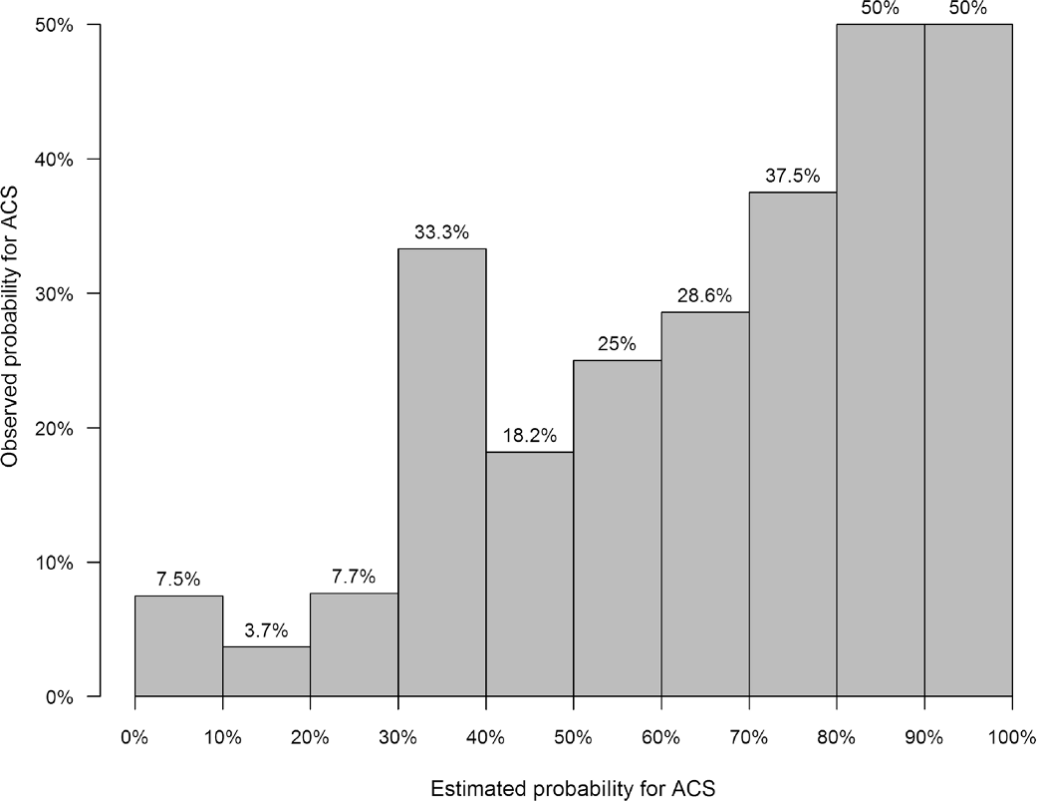
**Calibration plot showing observed versus predicted probabilities for ACS.**

Within 18 months 186 patients agreed to participate in the study. The median age of patients was 52 years (IQR 35-67), 130 (70%) of patients had male gender. In 168 (90%) patients the chief complaint was acute chest pain, and in 18 (10%) it was acute dyspnea, in 49 (26%) patients both symptoms were present at presentation in the emergency room (ER). In 42 patients troponin levels were elevated at the initial exam. Not myocardial ischemia only but infections, atrial fibrillation, heart failure, pulmonary embolism and other illnesses can cause an increase of troponin. So the number of patients with increased troponin levels is higher than the number of patients with myocardial ischemia. Further details about patient characteristics are shown in Table 1.In 31 (17%) patients an ACS was diagnosed, all of them have been hospitalized. In the two weeks after attending the emergency room in no further patient an ACS occurred. In eleven of the predictor variables of the prediction model there was a small percentage of missing values (range 1% to 9% of observations, median = 3%). The missing values were multiply imputed and the calculated probabilities of an ACS ranged from 0 to 1, median probability was 0.20 (IQR: 0.05-0.46). The calculated probabilities for more than one third of patients (36%) were less than 10%. The distribution of calculated versus observed probabilities is shown in Figure 1.

**Table 1:** **Characteristics of included patients with medical history, signs, symptoms, ECG and lab results**

Patient characteristics	Median/number	IQR/%
Time since onset of symptoms (h)	Median: 5	IQR: 2-30
Duration of symptoms (min)	Median: 180	IQR: 75-780
Acute dyspnea	67	36%
Acute chest pain	168	90%
Radiating chest pain	75	40%
Prior angina and acute episode provoked by lesser exertion and/or lasting longer	44	24%
Age (years)	Median: 52	IQR: 35-67
Male gender	130	70%
Body Mass Index in kg/m^2^	Median: 25.1	IQR: 22.4-28.4
Hypertension	83	45%
Smoking, number of cigarettes per day in month prior to episode	Median: 0	IQR: 0-5
Smoking, number of pack years	Median: 0	IQR: 0-11.5
Hypercholesterolemia	52	28%
Diabetes mellitus	24	13%
Peripheral vascular disease	9	5%
Stroke in personal history	2	1%
Myocardial infarction in personal history	35	19%
Heart rate (beats/minute)	Median: 70	IQR: 62-83
Mean blood pressure*	Median: 108.5	IQR: 99.9-118.5
ST elevation/depression ≥1 mV	20	11%
Hyperacute T wave	8	4%
Elevated troponin level	42	23%

*Mean blood pressure:= mean between systolic and diastolic blood pressure.

**Figure 2:**
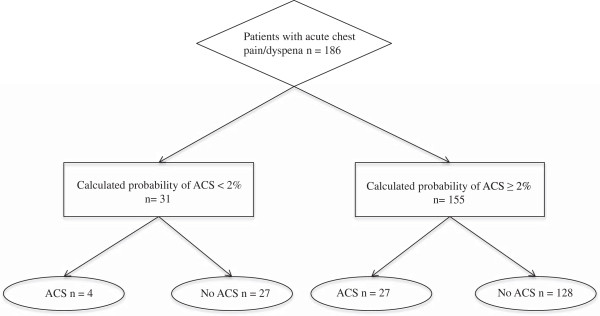
**Patient flow chart.** (ACS = Acute Coronary Syndrome).

Of particular interest is the performance of the prediction rule when the calculated probabilities for ACS are below 2% (Figure 2). Two percent probability of presence of ACS seems to be the rule-out threshold for physicians without further testing [5]. In 31 (17%) patients the calculated probability was less than 2% and four of them actually had an ACS resulting in a sensitivity of 87%, with 95% confidence interval (CI) 70%-96% and a specificity of 17%, 95% CI 12%-24% (Table 2). The positive predictive value (PPV) is 0.17 (95% CI 0.15-0.19) and the negative predictive value (NPV) is 0.88 (95% CI 0.73-0.95). The positive likelihood ratio LR + = 1.04 and the negative likelihood ratio LR- = 0.76. Further investigation in the four patients with ACS but predicted probability below 2% of it led to the finding that three patients had extraordinary long times since onset of symptoms: the values were 24 hours, 48 hours and 96 hours, respectively. The range of time since onset of symptoms was 1-9 hours in the vignettes used to derive the probability function. The fourth patient had an extremely large number of pack years smoked, which was 180 and for that reason also far outside of the range of values in the original developmental set of vignettes.

**Table 2:** **Diagnostic accuracy of the prediction rule for ACS**

Two-by-two table of observed versus predicted ACS			
Rule	ACS present	ACS absent	Sum
Pred. probability ≥2% (ACS pos.)	27	128	**155**
Pred. probability <2% (ACS neg.)	4	27	**31**
Sum	31	155	186
**Number of ACS cases versus deciles of predicted probability**			
**Predicted probability of ACS**	**Number of patients**	**Number of ACS cases**	
0% - <10%	67	5	
10% - <20%	27	1	
20% - <30%	26	2	
30% - <40%	12	4	
40% - <50%	11	2	
50% - <60%	8	2	
60% - <70%	7	2	
70% - <80%	8	3	
80% - <90%	10	5	
90% - <100%	10	5	

## Discussion

In a sample of patients in emergency rooms with chest pain and/or dyspnea the calculated probabilities, based on the diagnostic probability function, in general, overestimate the presence of ACS. In the intermediate probability range a certain degree of imprecision is without any negative consequences for the patient anyway because further tests to either rule-in or rule-out of ACS will be done. Relevant is a high accuracy in the extreme probability ranges to securely rule-out or rule-in ACS. From a clinician’s point of view a diagnostic aid to reliably rule out ACS would be of great help, because the majority of patients with chest pain and/or dyspnea do not have ACS. Desirable, according to a survey among physicians in Canada, would be a diagnostic aid that correctly identifies patients with a probability of ACS of less than two percent [5]. The threshold is that low because missing an ACS could be life threatening for the patient and delayed treatment might have a negative effect on the future course of the illness. Our study differs from other validation studies in two aspects. Firstly, the original derivation study is based on a prediction model derived from expect knowledge rather than from patient data. Secondly the derivation study aimed at generating a rule-out criterion for ACS.

In about every fifth participant of our study the calculated probabilities were less than two percent. In four of these 31 patients an ACS was present. A detailed assessment of the patient characteristics revealed that all of the four patients were rather different with respect to two predictor variables compared to those from which the diagnostic probability has been derived. In the derivation sample the time since onset of symptoms ranged from one to nine hours, and in three patients of the validation sample the time since onset was more than 24 hours, in the extreme case 4 days. In the derivation sample the number of pack-years ranged from 0 to 40 pack-years and the patient of the validation sample smoked 180 pack-years. The estimated effect of the predictors in the risk model is strongly dependent on the predictors’ ranges in the derivation sample. For that reason one would expect to obtain less precise results if the validation sample differs from the derivation sample. A solution for this problem could be to update the original prediction model to include patients with longer duration of symptoms and larger number of pack-years [7].

### Comparison to literature

Pozen et al. [8] were the first to develop a diagnostic aid to calculate the patient’s probability of a myocardial infarction in a patient with acute chest pain. Since then further instruments have been developed and validated in different patient populations [9]. Among those validation studies of diagnostic instruments for ruling out ACS, sensitivity varied between 40 and 99%, whereas the range for specificity was 4 to 90%. The diagnostic aid with the highest value for securely ruling-out was developed by Selker et al. [10]. The derivation study that we intended to validate differs from other published prediction rules as it is intended to be used as a rule-out criterion. Therefore a direct comparison of our results with other published prediction rules is impossible since no other study applied a cut-off value as low as 2% predicted probability.

### Limitations and strengths

We have to point out some limitations of this study. First, patients were probably not enrolled consecutively and it might be that patients with a high suspicion of ACS were more likely to be included. Supporting evidence for this assumption is the fact that the prevalence for ACS was 17% in the sample, a number that is in the range of other studies [9]. A further limitation of our prediction rule may be that a relatively large number of variables is needed, and with a computer calculating the predicted probabilities of ACS. In the age of electronic patient charts and clinical information systems the information should be easy to extract and the calculations will be straightforward.

A strength of the study is that in all patients at least one further ECG and blood exam was performed within three to six hours after the first examination. All patients without diagnosis of ACS in the emergency room were contacted and interviewed by telephone ten to fourteen days after a visit of the emergency room. We cannot exclude that patients were diagnosed with cardiovascular disease later. However, the current study focused on the safety of emergency department assessment for acute coronary syndrome.

### Clinical implications

Even though only a minority of patients with chest pain has ACS or other life-threatening diseases, clinicians in the emergency room are cautious when making decisions about patients with chest pain. Cumulative data from the Physicians Insurers Association of America from 1985 to 2010 indicate that in emergency medicine chest pain is among the most prevalent and most expensive conditions for malpractice claims in the United States [11]. As a consequence, clinicians initiate diagnostic testing for ACS at very low threshold levels. Current evaluation strategies typically require clinical observation for several hours, serial electrocardiograms and biomarker measurements and eventually some form of functional testing to exclude myocardial ischemia. As emergency room clinicians are under considerable pressure to reduce resource use without increasing the probability of adverse outcomes in patients with chest pain a reliable instrument to rule out ACS after a first examination would be of great value.

### Future research

It appears that the domain of the derivation study was drawn too narrow for some of the patients with acute onset of chest pain and/or dyspnea. The estimated coefficients for duration of symptoms and number of pack years cannot be extrapolated to values far outside the range of the original vignettes. Therefore future research should address the question of how to derive a new or updated diagnostic probability function with wider ranges of continuous predictors. After that, the new probability function should be validated in a consecutive sample of patients.

The results of our study also have implications for the derivation of new prediction rules in other clinical applications based on expert knowledge. As described above, we found that our rule performed less than optimal if continuous predictors were far outside the original range of values. If broad ranges of values in the derivation study do not seem reasonable in the first place, future studies should include some kind of sensitivity analysis for the rule’s out-of-sample prediction performance.

## Conclusion

The proposed probability function, with a 2% cut-off for ruling out ACS works quite well if the patient data lie within the ranges of values of the original vignettes. If the observations deviate too much from these ranges, the predicted probabilities for ACS should be seen with caution.

### Ethical approval

The study has been approved by the ethical committees of the Swiss Cantons St. Gallen and Zurich (EKSG 10/067/1B and KEK-ZH-NR:2010-0058/0).

## Electronic supplementary material

Additional file 1: **Statistical variates, description and risk probability function.** (DOCX 17 KB)
