# Supplementary material for: Validation of a diagnostic probability function for estimating probabilities of acute coronary syndrome
Source: BMC Emerg Med. 2014 Nov 18;14:23. doi: 10.1186/1471-227X-14-23 (PMC4289321; doi:10.1186/1471-227X-14-23)
Supplement: Supplementary file 1 — Additional file 1: Statistical variates, description and risk probability function. (DOCX 17 KB) [file 12873_2013_215_MOESM1_ESM.docx]

**Appendix**:

Statistical variates, description and risk probability function.

| Variate | Description |
| --- | --- |
| X1 | log of time, in hours, since onset of symptoms |
| X2 | (X1)^2 |
| X3 | log of duration of symptoms in minutes |
| X4 | (X3)^2 |
| X5 | Indicator of dyspnea |
| X6 | Indicator of chest pain |
| X7 | Indicator of burning type of chest pain |
| X8 | Indicator of pressure/tightness type of chest pain |
| X9 | Indicator of aggravation of chest pain by inspiration or change of position |
| X10 | Indicator of radiation of chest pain |
| X11 | Indicator of nausea during episode |
| X12 | Indicator of diaphoresis during episode |
| X13 | Indicator of dizziness during episode |
| X14 | Indicator of fever just prior to episode |
| X15 | Indicator of leg pain just prior to episode |
| X16 | Indicator of angina prior to present chest pain |
| X17 | Indicator of prior angina provoked by lesser exertion and/or lasting longer |
| X18 | Indicator of palpitations in days prior to episode |
| X19 | Smoking, number of cigarettes/day, in month before episode |
| X20 | (X19)^2 |
| X21 | Indicator of respiratory infection during prior two weeks |
| X22 | Indicator of physical exertion immediately prior to episode |
| X23 | Indicator of emotional stress immediately prior to episode |
| X24 | Indicator of cocaine use immediately prior to episode |
| X25 | Age in years |
| X26 | (X25)^2 |
| X27 | Indicator of male gender |
| X28 | Body Mass Index in kg/m^2 |
| X29 | (X28)^2 |
| X30 | Smoking, number in pack-years |
| X31 | (X30 -10)^2 |
| X32 | Indicator of doctor recommendation for antihypertensive treatment |
| X33 | Indicator of doctor recommendation for lipid lowering treatment |
| X34 | Indicator of doctor diagnosis of diabetes |
| X35 | Indicator of doctor diagnosis of peripheral vascular disease |
| X36 | Indicator of doctor diagnosis of stroke |
| X37 | Indicator of doctor diagnosis of myocardial infarction |
| X38 | Indicator of pale skin |
| X39 | Indicator of irregular pulse |
| X40 | Heart rate in beats/minute |
| X41 | (X40)^2 |
| X42 | Mean blood pressure |
| X43 | Indicator of heart failure at physical examination |
| X44 | Indicator of pericardial rub at physical examination |
| X45 | Indicator of chest pain aggravated by pressure on chest at physical examination |
| X46 | Indicator of difference in leg circumference at physical examination |
| X47 | ST elevation/depression in mV |
| X48 | (X47)^2 |
| X49 | Indicator of hyperacute T |
| X50 | Indicator of arrhythmia, left bundle branch block, and/or T inversion |
| X52 | Indicator of elevated troponin |
| X53 | X1 x X47 |
| X54 | X2 x X47 |

Risk probability function for individual patient data (shrinked regression coefficients):

S = 2.656311 – 0.118153 X1 + 0.016328 X2 + 0.925159 X3 – 0.114328 X4 + 0.472391 X5 + 0.685865 X6 – 0.1574 X7 + 0.307666 X8 + 0.275493 X9 + 0.319744 X10 + 0.106311 X11 + 0.335969 X12 + 0.216219 X13 – 0.678756 X14 – 0.169789 X15 – 0.081444 X16 + 0.754905 X17 – 0.02806 X18 – 0.024371 X19 + 0.000821 X20 – 0.355736 X21 + 0.202604 X22 + 0.221014 X23 + 0.592444 X24 + 0.000285 X25 + 0.000044 X26 + 0.296019 X27 – 0.539706 X28 + 0.01048 X29 + 0.014495 X30 – 0.003566 X31 – 0.052717 X32 + 0.101621 X33 + 0.299495 X34 + 0.301939 X35 + 0.165363 X36 + 0.692688 X37 + 0.227408 X38 – 0.123176 X39 + 0.006428 X40 – 0.00002 X41 – 0.006616 X42 + 0.30081 X43 – 1.017683 X44 – 0.042212 X45 – 0.149536 X46 – 1.014589 X47 + 0.995598 X48 + 1.069417 X49 + 0.180439 X50 + 1.960667 X52 + 1.017969 X53 – 0.538077 X54

The value S needs to be transformed to probability scale with the antilogit function:

P = exp(S) / (1+exp(S)),

resulting in a probability P for ACS.
